# Supplementary material for: The Optimal Retinal Locus for High-Resolution Vision in Space and Time
Source: bioRxiv. 2025 May 6:2025.04.30.650879. Preprint. [Version 1] doi: 10.1101/2025.04.30.650879 (PMC12247686; doi:10.1101/2025.04.30.650879)
Supplement: 1 [file NIHPP2025.04.30.650879V1-supplement-1.pdf]

bioRxiv preprint doi: https://doi.org/10.1101/2025.04.30.650879; this version posted May 6, 2025. The copyright holder for this preprint (which was not certified by peer review) is the author/funder, who has granted bioRxiv a license to display the preprint in perpetuity. It is made available under aCC-BY 4.0 International license.

Supplementary Materials for

The Optimal Retinal Locus for High-Resolution Vision in Space and Time

Josselin Gautier *et al*

*Corresponding author. email: aroorda@berkeley.edu

This PDF file includes:

Figs. S1-S5

Legend for Movies S1-S2

Other Supplementary Material for this manuscript includes the following:

Movies S1-S2

bioRxiv preprint doi: https://doi.org/10.1101/2025.04.30.650879; this version posted May 6, 2025. The copyright holder for this preprint (which was not certified by peer review) is the author/funder, who has granted bioRxiv a license to display the preprint in perpetuity. It is made available under aCC-BY 4.0 International license.

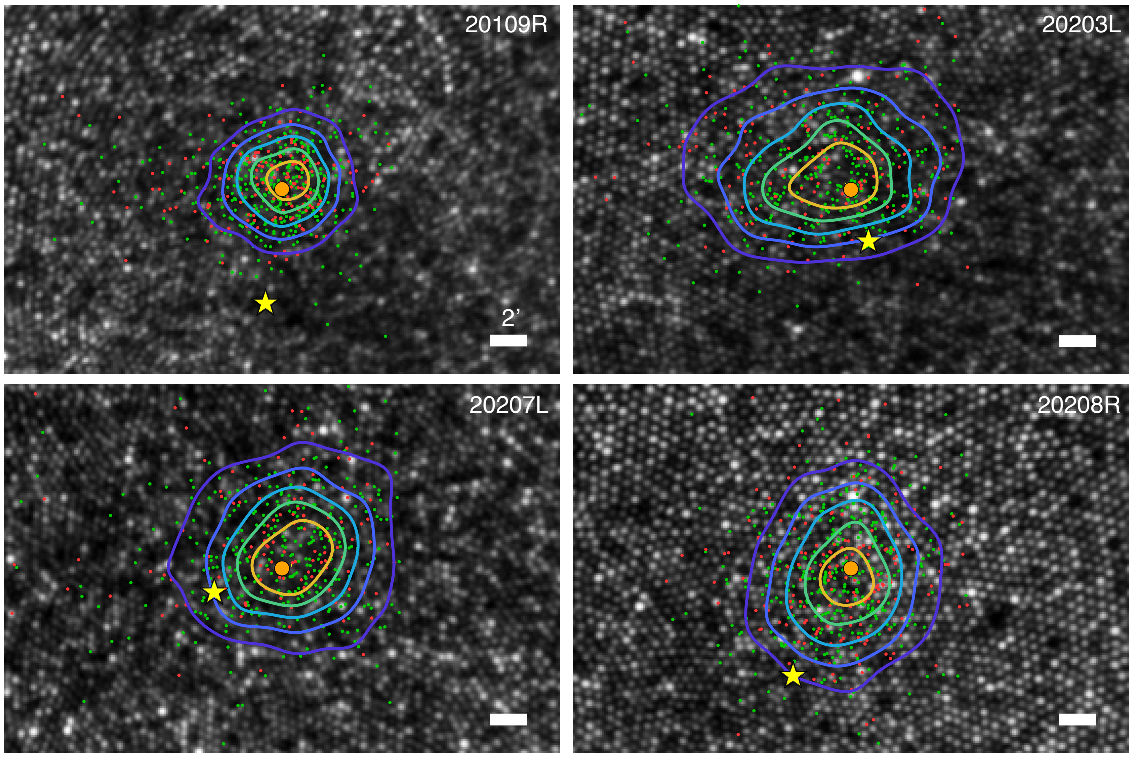

Figure S1: **Stimulus positions and performance for the four subjects not shown in Fig. 2B.** The position of the stimulus is plotted for each subject. Green and red dots represent those positions on trials in which the response was correct and incorrect, respectively. Orange circles represent the PRL. Yellow stars represent the cone-density centroids (CDCs). The colored contours indicate, respectively, 17% of positions falling closed to the PRL (yellow), 18–33% (green), 34–50% (cyan), 51–67% (blue), and 68–83% (purple).

bioRxiv preprint doi: https://doi.org/10.1101/2025.04.30.650879; this version posted May 6, 2025. The copyright holder for this preprint (which was not certified by peer review) is the author/funder, who has granted bioRxiv a license to display the preprint in perpetuity. It is made available under aCC-BY 4.0 International license.

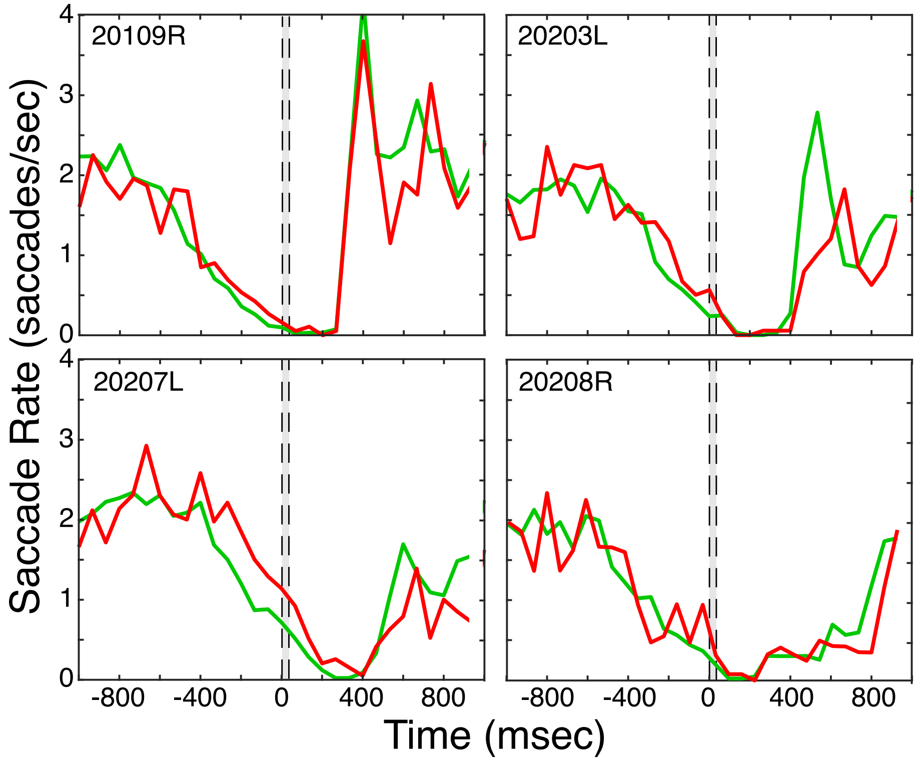

Figure S2: **Saccade rate over time. The average number of saccades per second is plotted as a function of time for the four subjects not shown in Fig. 3A.** Stimulus onset is at time 0. The green and red lines represent saccade rate during trials in which the observer responded correctly and incorrectly.

bioRxiv preprint doi: https://doi.org/10.1101/2025.04.30.650879; this version posted May 6, 2025. The copyright holder for this preprint (which was not certified by peer review) is the author/funder, who has granted bioRxiv a license to display the preprint in perpetuity. It is made available under aCC-BY 4.0 International license.

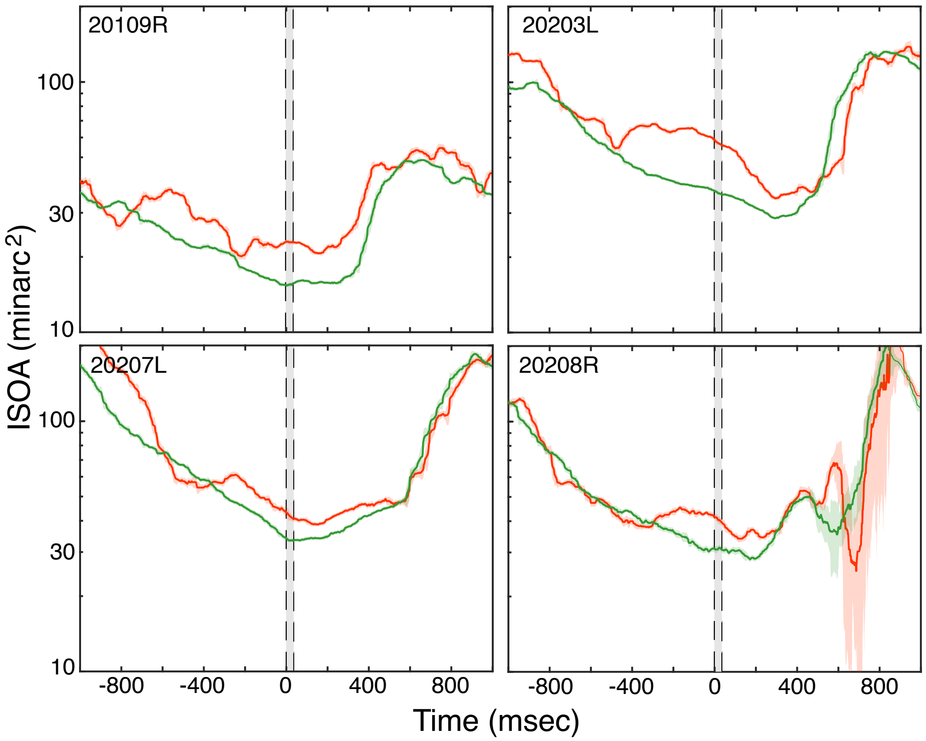

Figure S3: **ISO area over time for the four subjects not shown in Fig. 4A.** The area of the ISO contour containing 68% of retinal positions is plotted over the 2sec duration of a trial. The time at which the stimulus was presented is represented by the gray bar. The data have been averaged across the 2100 trials the subjects encountered. For each time a running median was computer over the 20 preceding and 20 succeeding time samples. The green and red lines represent contour areas on correct and incorrect trials, respectively. The shaded regions around each line indicate standard deviations.

bioRxiv preprint doi: https://doi.org/10.1101/2025.04.30.650879; this version posted May 6, 2025. The copyright holder for this preprint (which was not certified by peer review) is the author/funder, who has granted bioRxiv a license to display the preprint in perpetuity. It is made available under aCC-BY 4.0 International license.

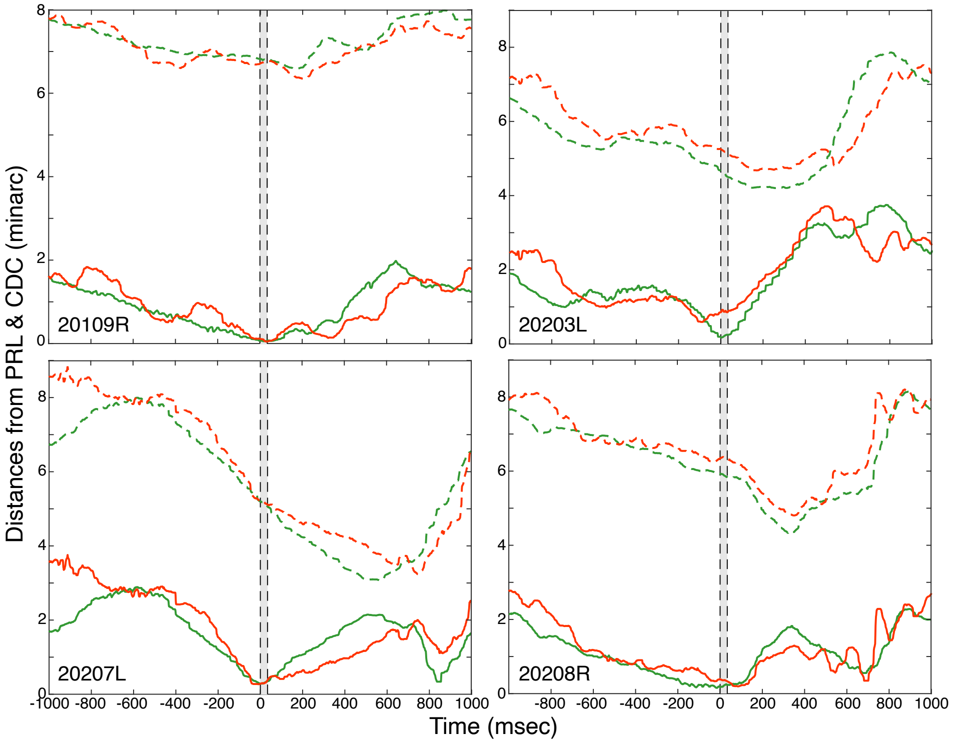

Figure S4: **Fixation relative to PRL and CDC during the course of a trial for the four subjects not shown in Fig. 4B.** The solid lower contours represent the median distance of fixation from the PRL the duration of a trial. The time at which the stimulus was presented is represented by the gray bar. The data have been averaged across the 2100 trials the observers encountered. The green and red lines represent distance from PRL on correct and incorrect trials, respectively. The dashed upper contours represent the median distance of fixation from the CDC. Again green and red represent distance on correct and incorrect trials, respectively.

bioRxiv preprint doi: https://doi.org/10.1101/2025.04.30.650879; this version posted May 6, 2025. The copyright holder for this preprint (which was not certified by peer review) is the author/funder, who has granted bioRxiv a license to display the preprint in perpetuity. It is made available under aCC-BY 4.0 International license.

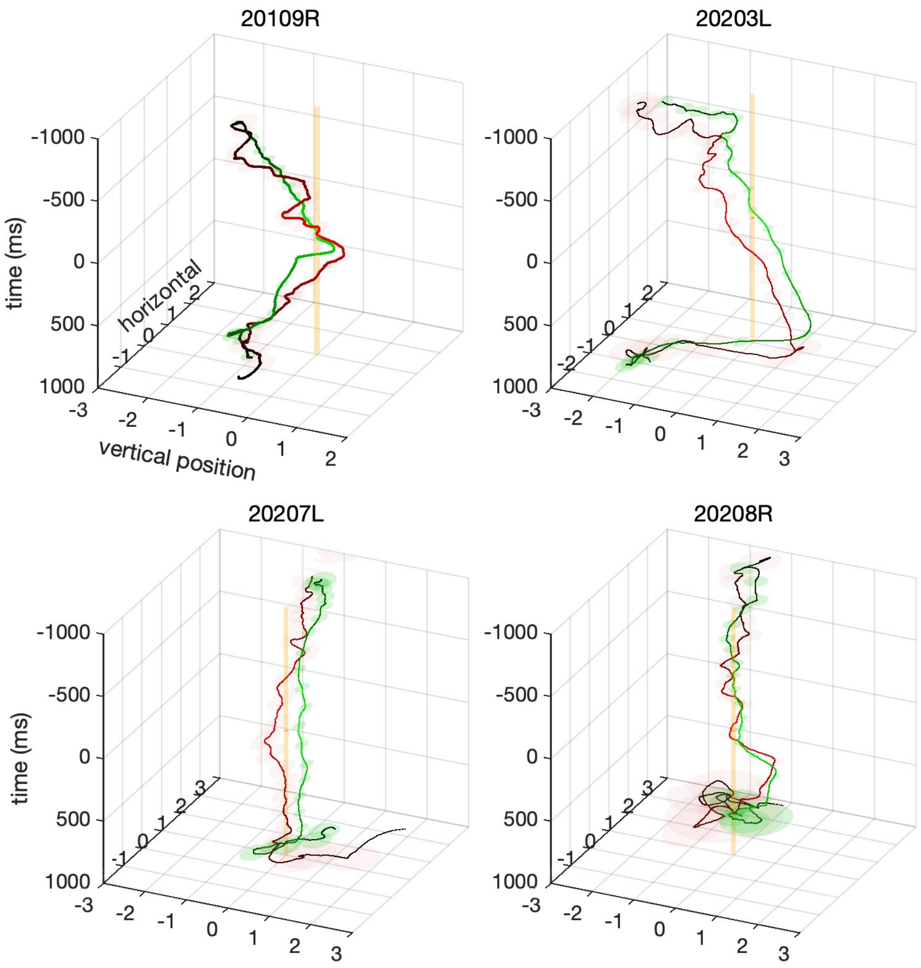

Figure S5: **Median eye trajectories over the course of the 2-sec trials for the four subjects not shown in Fig. 5.** The 3D coordinates are horizontal position and vertical position in minarc and time in msec. A 3D coordinate was calculated for every time sample in each trial. The median of those points was computed for each time sample and contours were drawn from those points using a Savitzky-Golay filer to smooth the data. The green and red traces are the trajectories on correct and incorrect trials, respectively. The transparent green and red ellipses represent horizontal and vertical position variability every 76msec. Trials in which the Vernier gap was +18secarc have been excluded because performance was at ceiling on those trials. Including them does not, however, affect these trajectories noticeably. The orange cylinders represent the fPRL. An animation of the 3D plot is shown in Supplemental Materials Movie S2.

bioRxiv preprint doi: https://doi.org/10.1101/2025.04.30.650879; this version posted May 6, 2025. The copyright holder for this preprint (which was not certified by peer review) is the author/funder, who has granted bioRxiv a license to display the preprint in perpetuity. It is made available under aCC-BY 4.0 International license.

Movie S1. Animation of 3D plot from Fig. 5

Movie S2. Animation of 3D plot from Fig. S5
